# Altered Brain Adiponectin Receptor Expression in the 5XFAD Mouse Model of Alzheimer’s Disease

**DOI:** 10.3390/ph13070150

**Published:** 2020-07-12

**Authors:** Anishchal A. Pratap, R. M. Damian Holsinger

**Affiliations:** 1 Laboratory of Molecular Neuroscience and Dementia, Brain and Mind Centre, Faculty of Medicine and Health, The University of Sydney, Sydney, NSW 2050, Australia; apra8538@uni.sydney.edu.au; 2 Discipline of Pathology, School of Medical Sciences, Faculty of Medicine and Health, The University of Sydney, Sydney, NSW 2006, Australia

**Keywords:** adiponectin receptors, astrocytes, Alzheimer’s disease, glia, metabolic dysregulation, neurodegenerative disease, neuroinflammation

## Abstract

Metabolic syndromes share common pathologies with Alzheimer’s disease (AD). Adiponectin, an adipocyte-derived protein, regulates energy metabolism via its receptors, AdipoR1 and AdipoR2. To investigate the distribution of adiponectin receptors (AdipoRs) in Alzheimer’s, we examined their expression in the aged 5XFAD mouse model of AD. In age-matched wild-type mice, we observed neuronal expression of both ARs throughout the brain as well as endothelial expression of AdipoR1. The pattern of receptor expression in the aged 5XFAD brain was significantly perturbed. Here, we observed decreased neuronal expression of both ARs and decreased endothelial expression of AdipoR1, but robust expression of AdipoR2 in activated astrocytes. We also observed AdipoR2-expressing astrocytes in the dorsomedial hypothalamic and thalamic mediodorsal nuclei, suggesting the possibility that astrocytes utilise AdipoR2 signalling to fuel their activated state in the AD brain. These findings provide further evidence of a metabolic disturbance and demonstrate a potential shift in energy utilisation in the AD brain, supporting imaging studies performed in AD patients.

## 1. Introduction

Alzheimer’s disease (AD) is a neurodegenerative disorder that represents approximately 60–80% of all cases of dementia [1]. Aging is by far the most prevalent risk factor for the development of AD, with 95% of patients being diagnosed over the age of 65 [2]. These cases have no clear aetiology, nor mechanisms of onset. However, the pathologies associated with AD include the presence of amyloid plaques comprised of amyloid-β (Aβ) aggregates [3,4,5,6,7,8,9,10], neurofibrillary tangles resulting from the intra-neuronal accumulation of hyperphosphorylated tau protein [11], and neuroinflammation that causes gliosis and further exacerbates Aβ production [12,13,14,15].

Alzheimer’s is a multifactorial disease with various genetic, environmental, and epigenetic factors that can lead to excessive accumulation of Aβ in the brain. Diet and exercise are modifiable risk factors for preventing and delaying the onset of AD [1,16,17]. Unhealthy diets and a lack of exercise can cause metabolic syndromes, such as obesity, type 2 diabetes mellitus, hypercholesterolemia, hypertension, and atherosclerosis, which may create a knock-on effect leading to AD-associated pathologies later in life [18,19,20,21,22].

It has been proposed that an imbalance in cerebral metabolism may in fact be the missing link between increased inflammation, insulin resistance, and cortical atrophy [23,24,25]. In obesity, hypertrophy of adipose tissue leads to a proportional increase in secreted adipokines [26]. Adipose tissue is instrumental in the development of metabolic syndromes, due to an overproduction of leptin, tumour necrosis factor (TNF), interleukin (IL)-6, IL-10, and reduced production of adiponectin [27,28,29,30]. Numerous adipokines cross the blood–brain barrier (BBB) and, in turn, act on hypothalamic neurons to mediate energy expenditure [31]. As such, the actions of adipokines in the central nervous system (CNS) and, specifically, their effects in neurological diseases, such as AD, are of significant interest. 

Adiponectin is a large (30 kDa) adipokine molecule secreted by adipocytes and displays protective roles against many diseases and conditions, including atherosclerosis [32], inflammation [33], various types of cancer [34], and insulin resistance, through its receptors AdipoR1 and AdipoR2 [35,36,37]. Individuals who are obese and/or diabetic have decreased adiponectin in circulation, in addition to hyperglycaemia, dyslipidaemia, and hyperinsulinemia [38]. AdipoR1 and AdipoR2 are differentially expressed in the body, but both are expressed in the CNS [39]. Deletion of the AdipoR1 gene in the db/db (leptin receptor mutation) mouse model of diabetes and obesity resulted in decreased adenosine monophosphate-activated protein kinase (AMPK) activation, thereby reducing glucose uptake [40]. Deletion of AdipoR2 resulted in decreased peroxisome proliferator-activated receptor alpha (PPARα) signalling, reducing fatty acid oxidation and energy production. Simultaneous deletion of both adiponectin receptors resulted in an increase in free fatty acids (FFAs), reactive oxygen species (ROS), and inflammation that all led to insulin resistance and diabetes [40]. This implies that activation of both AdipoR1 and AdipoR2 acts synergistically through their respective signalling pathways (AMPK and PPARα, respectively) to increase insulin sensitivity and decrease triglyceride content in circulation. Additionally, adiponectin knock-out mice exhibit neuroinflammation and neuronal and synaptic loss in the hippocampus and cerebral cortex, as well as cerebral insulin resistance [41], suggesting that decreased levels of adiponectin in the CNS could have detrimental effects on memory and cortical function. 

Adiponectin is also involved in metabolic dysregulation in AD patients [42]. Levels of adipokine in serum were higher in AD patients [43], but lower in cerebrospinal fluid (CSF) in patients with AD and mild cognitive impairment (MCI), compared to normal healthy controls [44]. A recent study reported that, while adiponectin levels were reduced in the brains of AD patients and 5XFAD mice, AdipoR levels were increased in both human and 5XFAD frontal cortices and hippocampi [45]. This represents a change in metabolic signalling as AD develops, resulting in altered adiponectin levels in serum and CSF, and AdipoR levels in the brain. Moreover, Kim and colleagues found that AdipoR1 suppression led to neurodegeneration in wild-type (C57BL-6J) mice [46]. The group also reported similar neuropathologies to those of AD, including memory impairment, increased Aβ, and phosphorylated tau load. This clearly demonstrates that the dysfunction of AdipoRs plays a role in AD neuropathology. While some research groups have attempted to shed light on the role of AdipoR1 and R2 in AD [45,46,47], the expression pattern of AdipoRs in transgenic AD mice is unknown. To address these questions, we explored the expression and distribution of AdipoR1 and AdipoR2 in the well-characterised 5XFAD mouse model of AD.

## 2. Results

### 2.1. AdipoR1 and AdipoR2 Is Expressed throughout the Mouse Cortex and Hippocampus

**Figure 1:**
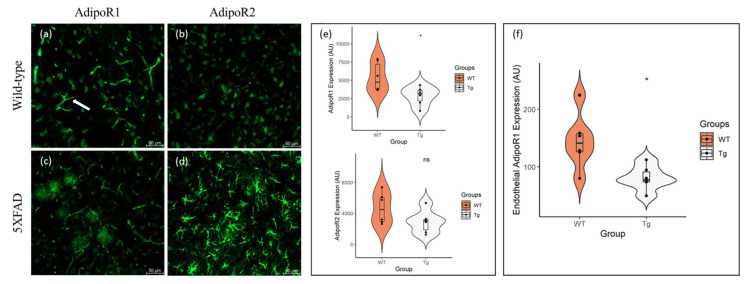
Expression of AdipoR1 and AdipoR2 in the cortices of 48–52-week-old 5XFAD and wild-type (WT) mice. AdipoR1 is present in neurons of both (**a**) WT and (**c**) 5XFAD mice, in addition to endothelial cells lining blood vessels (arrow). Both (**b**) WT and (**d**) 5XFAD mice also express AdipoR2 in neurons, with substantial glial expression of AdipoR2 in (**d**) 5XFAD mice. There was a reduction in (**e**) neuronal AdipoR1 and AdipoR2 expression in the 5XFAD (Tg) cortex, compared to controls, with statistical significance observed in AdipoR1 expression. Endothelial expression (**f**) of AdipoR1 was also significantly reduced in the 5XFAD (Tg) cortex. Data are presented as Mean ± SEM using independent t-test with statistical significance * *p* < 0.05 denoted between groups. Images were taken at 20× magnification. Scale bar = 50 μm.

Similar to previous reports [48], we found that AdipoR1 and AdipoR2 is expressed in neurons throughout the cortex of 48–52-week-old, aged wild-type (WT) and 5XFAD mice. Additionally, we observed different patterns of non-neuronal expression of AdipoR1 and AdipoR2. As previously reported [49], AdipoR1 is expressed by endothelial cells that constitute blood vessels (Figure 1a) and is seen throughout the brain. Contrarily, AdipoR2 is sparsely expressed by glial cells in the aged WT brain (Figure 1b).

We report reduced neuronal expression of AdipoR1 (Figure 1c) and AdipoR2 (Figure 1d) in the aged 5XFAD mouse. Quantification revealed a significant reduction in neuronal AdipoR1 expression in the 5XFAD cortex, compared with WT mice (t(10) = 2.656, *p* < 0.024, Cohen’s D = 1.53). Although there was a reduction in neuronal AdipoR2 expression in the AD mice, compared to age-matched controls, the difference was not statistically significant (t(10) = 2.656, *p* < 0.111, Cohen’s D = 1.00) (Figure 1e). 

We also observed a significant reduction in AdipoR1 expression in endothelial cells in the 5XFAD cortex, compared to aged WT mice (t(10) = 2.998, *p* < 0.013, Cohen’s D = 1.73) (Figure 1f). In addition, glial expression of AdipoR2 in the 5XFAD mouse was robustly increased when compared to controls. This may demonstrate a shift in metabolic expenditure from neurons to glia, whereby glial cells utilise AdipoR2 signalling to support their function of Aβ clearance in the AD brain. 

### 2.2. Astrocytes Express AdipoR2 in the 5XFAD Mouse

**Figure 2:**
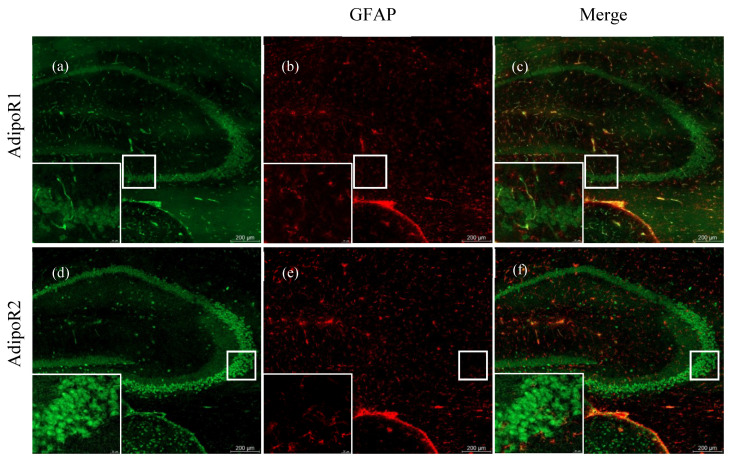
AdipoR and glial fibrillary acidic protein (GFAP) expression in hippocampi of 48–52-week-old wild-type mice. Hippocampal expression of glial fibrillary acidic protein (GFAP) in astrocytes of 5XFAD mice are shown in the middle panels (**b**,**e**). AdipoR1 is expressed in the hippocampus in both neuronal and endothelial cells (**a**). There is no colocalisation of AdipoR1 with GFAP in astrocytes (**c**). AdipoR2 is widely expressed in the hippocampal neurons (**d**), but it is not expressed in astrocytes (**f**). Images were acquired at 20× magnification. Scale bars for (**a**–**f**) are 200 μm. Scale bars for zoomed-in figures are 20 μm.

AdipoR1 (Figure 2a) and R2 (Figure 2d) are robustly expressed in the hippocampus of an aged WT mouse. AdipoR1 is also prevalent in endothelial cells of blood vessels throughout and surrounding the hippocampus (Figure 2a). There is minimal expression of activated astrocytes in the WT cortex, which is demonstrated by sparse expression of glial fibrillary acidic protein (GFAP) (Figure 2b,e), a marker of activated astrocytes. Moreover, AdipoR1 and AdipoR2 are not expressed by astrocytes in the WT hippocampus (Figure 2c,d).

**Figure 3:**
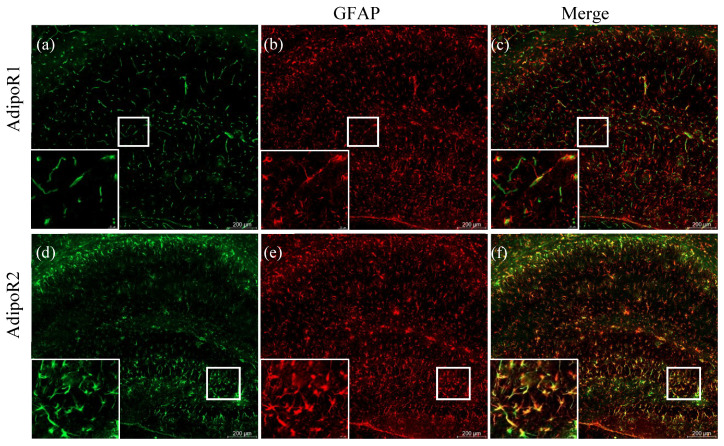
Astrocytic expression of adiponectin receptors in 48–52-week-old 5XFAD mice. Double labelling of AdipoR1 (**a**) with GFAP (**b**) showed little expression of AdipoR1 in astrocytes (**c**). Significant colocalisation (**f**) of AdipoR2 (**d**) with GFAP (**e**) demonstrates astrocytic expression of AdipoR2. Images were acquired at 20× magnification. Scale bars for (**a**–**f**) are 200 μm. Scale bars for zoomed-in figures are 20 μm.

In the 5XFAD mouse, amyloid plaque deposits begin to appear by 2 months of age and are accompanied by widespread neuroinflammation [50]. Similar to previous reports, we observed increased neuroinflammatory responses throughout the cortex and subcortical structures, including the hippocampus, which were replete with activated astrocytes (Figure 3b,e).

**Figure 4:**
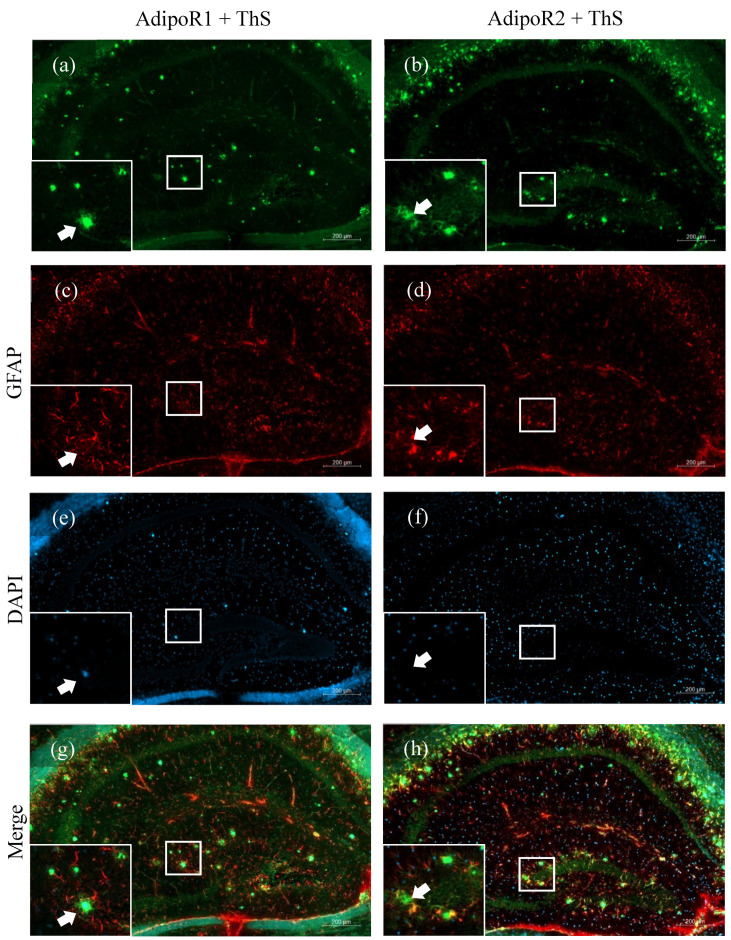
Amyloid plaque staining and adiponectin receptor expression in the hippocampi of 48–52-week-old 5XFAD mice. AdipoR1 (**a**) double-labelled with GFAP (**c**) and counterstained with 4′,6-diamidino-2-phenylindole (DAPI) (**e**) displayed no AdipoR1-expressing astrocytes surrounding Thioflavin-S-stained amyloid plaques (arrows) in the hippocampus (**g**). Astrocytes expressing AdipoR2 (**b**) double-labelled with GFAP (**d**) and counterstained with DAPI (**f**) can be seen surrounding amyloid plaques (arrows) (**h**). Images were acquired at 20× magnification. Scale bars for (**a**–**h**) are 200 μm. Scale bars for zoomed-in figures are 20 μm.

In the hippocampus, we observed increased glial expression of AdipoR2 (Figure 3d) and, to a much lesser extent, AdipoR1 (Figure 3a). To determine whether AdipoR1 and AdipoR2 were expressed by astrocytes, sections were double-labelled for adiponectin receptors and GFAP. We observed colocalisation of AdipoR1 and AdipoR2 with GFAP in astrocytes in the hippocampus of 5XFAD mice (Figure 3c,f). In AD, activated astrocytes surround amyloid plaques and release pro-inflammatory cytokines to degrade the accumulated toxic Aβ peptides. We show that astrocytes surrounding amyloid plaques robustly express AdipoR2 (Figure 4h), compared to AdipoR1 (Figure 4g), indicating a preference for the R2 receptor to fuel the metabolic needs of the activated astrocyte.

### 2.3. Increased Expression of AdipoR2 in Thalamic and Hypothalamic Areas

**Figure 5:**
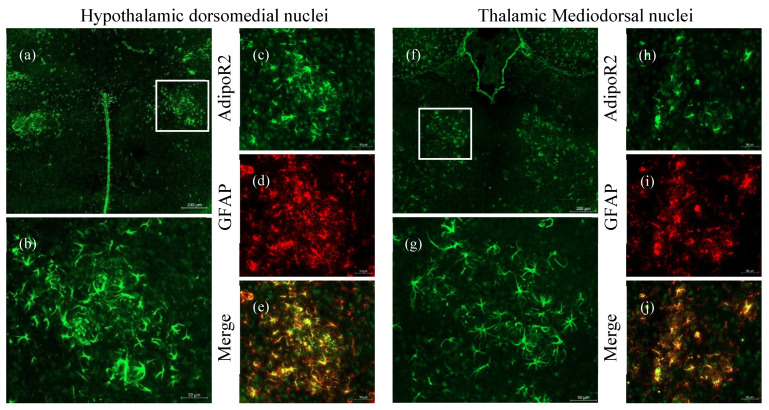
Astrocytes localised to the hypothalamic dorsomedial (DMHa) and thalamic mediodorsal (MD) regions in an aged 5XFAD mouse brain. Increased presence of AdipoR2 in the DMHa (**a**–**c**) and MD (**f**–**h**) in the thalamic nuclei of aged 5XFAD mice. Astrocytic expression of AdipoR2 (**e**,**j**) was confirmed by double-labelling with GFAP (**d**,**i**). Images were acquired at 20× magnification. Scale bars for (**a**) and (**f**) are 200 μm, (**b**–**e**) and (**g**–**j**) are 50 μm.

The hypothalamus is involved in various homeostatic functions. Areas including the hypothalamic dorsomedial nuclei (DMHa) and the thalamic mediodorsal nuclei (MD) are involved in homeostatic mechanisms including metabolic, cardiovascular, and gastrointestinal function [51]. In addition to the hippocampus and cortex, we also found activated astrocytes localised within the DMHa (Figure 5a–e) and MD (Figure 5f–j). These astrocytes displayed markedly increased AdipoR2 expression (Figure 5d,i). There were no activated astrocytes in the DMHa and MD of aged WT mice (Supplementary Figure S1), providing further evidence of metabolic impairment in AD.

## 3. Discussion

Metabolic and neurodegenerative diseases, including Alzheimer’s, display neuroinflammatory states that involve upregulation of pro-inflammatory cytokines, which, in turn, induce a reactive glial response [33,52,53]. Here, we examined the expression of the metabolic receptors of adiponectin, AdipoR1, and AdipoR2 in a mouse model of AD neuropathology. Adiponectin receptors are known to play a role in insulin sensitisation, upregulation of glucose intake, and reduction of inflammation through the AMPK and PPARα pathways, respectively [40]. Therefore, to determine whether these receptors were involved in metabolic dysregulation observed in AD, we sought to identify the expression patterns of AdipoR1 and AdipoR2 in the brains of aged 5XFAD mice.

Consistent with previous studies, we show that AdipoR1 and AdipoR2 are expressed in the mouse brain (Figure 1). Song and colleagues [49] observed decreased AdipoR1 expression in the 5XFAD mouse, but no significant difference in AdipoR2 expression, compared to WT littermates, using both immunohistochemical and western blotting techniques. Our findings add an additional level of detail to the expression patterns of adiponectin receptors. Using double immunofluorescence labelling, we report that in addition to the decreased expression of both AdipoR1 and AdipoR2 by neurons in the 5XFAD mouse brain compared to wild-type littermates, AdipoR1 is expressed by endothelial cells, and AdipoR2 is markedly increased in astrocytes. A recent study reported levels of AdipoRs in the frontal cortices and hippocampi of humans and 9-month-old 5XFAD mice [45]. Ng and colleagues observed increased expression of both AdipoR1 and R2 in both areas of the brain in AD patients and 5XFAD mice. Contrary to our findings, Ng and colleagues report increased expression of adiponectin receptors in neurons. There are many variables between the two studies that could account for these variations, including tissue processing techniques (Ng et al. used paraffin-embedded, proteinase-K-treated tissue, while we used fresh, frozen tissue) and age of mice (9 months in the Ng et al. study and 11–12 months in ours). Our ability to detect AdipoR expression in endothelial cells and astrocytes in the 5XFAD brain was only made possible using immunofluorescence labelling. Western blotting, as a technique, reveals whole tissue protein expression, but since the method requires tissue homogenisation, delineating cell-specific expression of proteins of interest is not feasible. Our results provide an interesting perspective on AdipoR expression in the 5XFAD mouse brain and provide insight into how these receptors may be involved in human AD pathology. Interestingly, Ng and colleagues reported that chronic, oral administration of an AdipoR agonist (AdipoRon) lowered plaque and Aβ levels in AD mice [45]. Our results strongly support these findings, as they would suggest that orally administered AdipoRon would fuel the astrocytes and facilitate their phagocytic activity, enabling the clearance of amyloid plaques and Aβ in the AD brain.

Accumulation of Aβ and hyperphosphorylated tau protein along with neuroinflammation are hallmark pathologies in AD [54]. In 5XFAD mice, early Aβ aggregation and deposition (by 8 weeks of age) further exacerbates the inflammatory state, thereby activating the resident glial cells, astrocytes, and microglia, in order to clear toxic amyloid build-up [55]. Astrocytes are important in Aβ removal as they express insulin-degrading enzyme (IDE), which is involved in the degradation of amyloid aggregates [56]. We report that reactive or activated astrocytes in the 5XFAD mouse brain also express high levels of AdipoR2. Astrocytes are involved in maintaining a homeostatic environment within the central nervous system (CNS). Additionally, these cells form a vital component of the blood–brain barrier (BBB), where astrocytic foot processes ensheath blood vessels and form a barrier that prevents leakage of material into the CNS. The increased expression of AdipoR2 in reactive astrocytes in the 5XFAD mouse brain could mediate access, via the PPARα pathway, to energy in the form of free fatty acids (FFA) in circulating blood, and fuel its increased activity in degrading aggregated amyloid.

An additional observation in our study was the astrocytic expression of AdipoR1 in 5XFAD mice. Although this expression was not as robust as that of AdipoR2, its presence in astrocytes marks another shift in expression and energy utilisation from neurons. Astrocytic expression of AdipoR1 has also been observed in a mouse model of intracerebral haemorrhage (ICH) [57]. This pattern of post-injury expression demonstrates that astrocytes may express adiponectin receptors when placed under high metabolic stress as an avenue to fuel its increased workload.

We also observed a significant reduction in neuronal expression of both AdipoR1 and AdipoR2 in the 5XFAD mouse brain. Cerebral insulin resistance is a well-known phenomenon in AD [18,19,20,23,53,58,59,60,61,62]. Impaired insulin signalling in neurons during disease states leads to deficits in glucose uptake and utilisation [63]. Since neurons have no mechanism of storing energy, a disruption in glucose acquisition would lead to debilitating effects on the cell. Adiponectin receptors are intimately involved in regulating insulin sensitivity and glucose uptake through the AMPK metabolic pathway [64]. Our data demonstrate a large shift in AdipoR2 expression from neurons to astrocytes in the AD brain and may represent a crosstalk between neurons and glia, whereby astrocytes “hijack” the available glucose to fuel the increased workload of degrading Aβ and protecting neurons.

We also discovered the presence of AdipoR2-expressing astrocytes in the hypothalamic dorsomedial nuclei (DMHa) and thalamic mediodorsal nuclei (MD) in 5XFAD mice (Figure 5). Interestingly, similar to cortical regions, neurons in these nuclei expressed little to no AdipoR2. The hypothalamus consists of groups of nuclei that maintain a myriad of functions, including energy and hormonal metabolism, in addition to circadian rhythm and sleep, among others. The DMHa nuclei in particular are important for feeding and circadian activity, while the MD mediate behaviour and arousal [51]. Disruption to body weight, systemic metabolism, sleep–wake cycle, and neuroendocrine secretions have been reported in AD [51,65,66]. These seemingly disparate manifestations may be linked to diencephalic dysfunction during various stages of AD, where the DMHa and MD may play a critical role in non-cognitive manifestations observed in AD [67,68]. Astrocytes are important mediators between systemic and cerebral environments. GFAP and AdipoR2 colocalisation in activated astrocytes in these nuclei may provide further insight into the role of these cells in disease processes whereby astrocytes utilise the AdipoR2 signalling pathway to fuel their reactive state to combat neuroinflammation. Our results provide evidence for the dysregulation of cerebral metabolism in the AD brain, as previously reported by imaging studies on AD patients [58,69,70]. The shift in the expression of AdipoR2 from neurons to astrocytes may also have a wider implication for diseases that include the activation of astrocytes. As such, future research should focus on other neuroinflammatory diseases that involve activated astrocytes.

## 4. Materials and Methods 

### 4.1. Chemicals and Reagents

The following primary antibodies were used for immunofluorescence experiments: Rabbit Anti-AdipoR1 (Cat# ab70362, RRID: AB_2221896), Rabbit Anti-AdipoR2 (Cat# ab231051, RRID: AB_2814663), and Mouse Anti-GFAP (glial fibrillary acidic protein) (Cat# ab10062, RRID: AB_296804). The secondary antibodies used in immunofluorescence included Goat Anti-Rabbit IgG H&L Alexa Fluor^®^ 488 (Cat# ab150077, RRID: AB_2630356) and Goat Anti-Mouse Alexa Fluor^®^ 594 (Cat# ab150116, RRID: AB_2650601). 4′,6-diamidino-2-phenylindole (DAPI) counterstain (Cat# D9542, Sigma-Aldrich) was used for nuclei staining.

### 4.2. Animals

For this series of experiments, we used the 5XFAD heterozygous (Het) mouse model of AD and their wild-type (WT) littermates. These mice express three human familial mutations for amyloid precursor protein (APP) (APP KM670/671NL (Swedish), APP I716V (Florida), and APP V7171 (London)), in addition to two presenilin 1 (PSEN1) mutations (PSEN1 M146L and PSEN1 L286V). To obtain Het mice, wild-type C57BL6 (WT) males were mated with Het females. Both male and female 5XFAD transgenic AD mice were used in this study due to their robust expression of amyloid plaques and cortical neurodegeneration from 2 months of age [71]. Mice were housed in cages together with WT littermates, were maintained on 12 h light–dark cycles, and had free access to water and a normal chow diet. A total of 12 animals (WT = 6 and 5XFAD = 6) were used in this study. Animals were housed at The University of Sydney and were bred under protocol AEC2016/964, and all procedures were in accordance with institutional guidelines.

### 4.3. Tissue Collection and Preparation

Mice were sacrificed under deep pentobarbital anaesthetic at 48–52 weeks of age, followed by decapitation. The brain was extracted, separated into cortex and cerebellum, mounted in Cryomatrix™ (Cat# 6769006, ThermoFisher Scientific) embedding medium, and snap frozen in liquid nitrogen. The tissues were stored at −80 °C until analysis.

### 4.4. Immunofluorescence

Tissues were sectioned at 16µm using a Leica CM1950 cryostat and stored on glass slides at −20 °C. Frozen sections were thawed at room temperature (RT) before being fixed in ice cold 100% methanol for 10 min. Post-fixation, sections were washed in PBS, followed by blocking in 1% goat serum (Cat# ab7481, RRID: AB_2716553) in phosphate buffered saline (PBS), and incubated in a humidity chamber for 30 min at RT. Sections were rinsed once again in PBS for 3 min, and the primary antibodies anti-AdipoR1 (1:300), anti-AdipoR2 (1:300), and anti-GFAP (1:1000) were applied to tissue sections and incubated overnight at 4 °C. The following day, sections were briefly washed with PBS and then incubated with either Goat Anti-Rabbit IgG H&L (Alexa Fluor^®^ 488) or Goat Anti-Mouse (Alexa Fluor^®^ 594) in a humidity chamber for 1 h at RT in the dark. Sections were washed with PBS, counterstained with DAPI (0.1 g/L), coverslipped with DPX mountant (Cat No. 06522, Sigma-Aldrich, Seven Hills, NSW, Australia), and stored at 4 °C prior to image analysis. The tissues were imaged using the Zeiss Axio Scan.Z1 slide scanner (Carl Zeiss, Oberkochen, Germany).

### 4.5. Thioflavin-S Staining

Following the last PBS wash in the immunofluorescent staining above, Thioflavin-S (ThS) in 50% ethanol (*w*/*v*) was added to each section and incubated in the dark for 10 min. The slides were then dehydrated in 3 washes of ethanol (80%, 80%, and 95% ethanol), and then rehydrated in three subsequent washes with double-distilled water before coverslipping. The slides were imaged using the Zeiss Axio Scan.Z1 slide scanner (Carl Zeiss, Oberkochen, Germany) as described above.

### 4.6. Quantification Analysis

Quantification analysis was performed using ImageJ (ImageJ, National Institutes of Health, USA). Briefly, ten random neuronal cells per sample were selected using the freehand tracing tool, and their fluorescence intensities were acquired. Background intensity represented ten regions that were adjacent to, but devoid of, cells. Data were acquired using the measure function on ImageJ and all data points, including area, mean, IntDen, and RawIntDen, were compiled in Microsoft Excel (Microsoft Corporation Inc., Washington, USA). A combined total cellular fluorescence (CTCF) value was determined by subtracting the background intensity from those acquired for the cells and averaging the values per sample (CTCF = integrated density − (mean area of cells × mean background intensities)) [72,73]. A similar method was used to quantify endothelial AdipoR1 expression by drawing an ROI (region of interest) line through the diameter of three endothelial cells per sample, and the background intensities obtained were from areas adjacent to the endothelial cells. The analysis for AdipoR1 endothelial CTCF utilised the same methods outlined above.

### 4.7. Statistical Analysis

An independent sample t-test was used to determine significance between the 5XFAD (*n* = 6) and age-matched controls (*n* = 6), with significance indicated at * *p* < 0.05. Statistical analysis was conducted using SPSS version 25 (IBM, New York, NY, USA) and R software.

## 5. Conclusions

We report an altered metabolic environment in the brains of the 5XFAD mouse model of Alzheimer’s disease, where we observe significantly decreased neuronal expression of the adiponectin receptors AdipoR1 and AdipoR2, and overexpression of AdipoR2 in activated astrocytes. Our results demonstrate that during the disease process, astrocytes robustly upregulate AdipoR2 expression, which we posit is tasked with fuelling these cells for the mission of degrading amyloid plaques and protecting neurons from a toxic milieu of neuroinflammation but indirectly leads to energy deficits in neurons and neuronal death in AD.

## Supplementary Materials

The following are available online at https://www.mdpi.com/1424-8247/13/7/150/s1, Figure S1: Hypothalamic dorsomedial (DMHa) and thalamic mediodorsal (MD) regions expressing AdipoR2 in wild-type mouse cortex. AdipoR2 is expressed by neurons in the DMHa (a, b) and MD (c, d) nuclei in aged WT mice at 48–52 weeks.
