# Supplementary material for: Altered Brain Adiponectin Receptor Expression in the 5XFAD Mouse Model of Alzheimer’s Disease
Source: Pharmaceuticals (Basel). 2020 Jul 12;13(7):150. doi: 10.3390/ph13070150 (PMC7407895; doi:10.3390/ph13070150)
Supplement: Supplementary file 1 [file pharmaceuticals-13-00150-s001.pdf]

Supplementary Figure 1

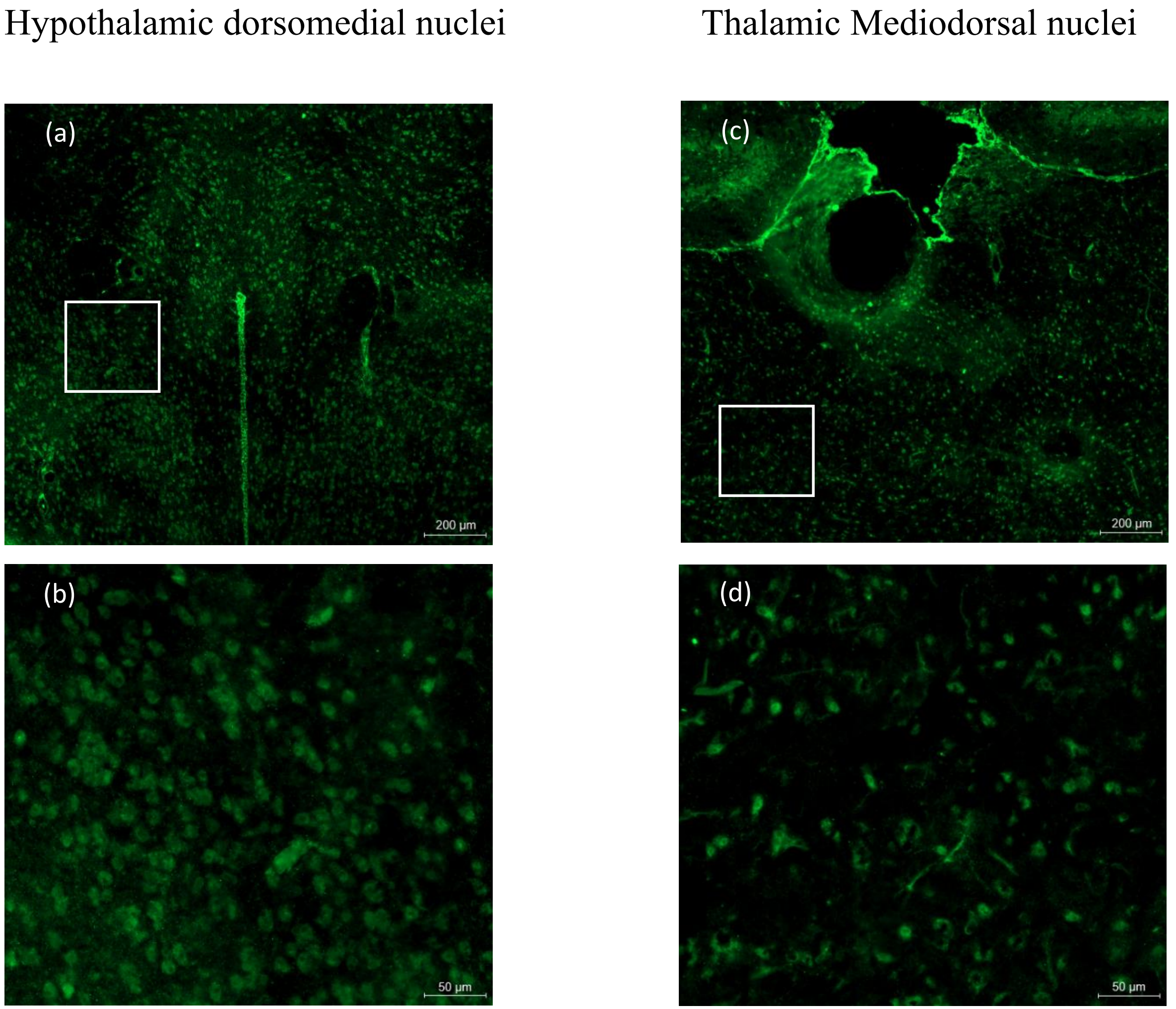

Supplementary Figure 1: Hypothalamic dorsomedial (DMHa) and thalamic mediodorsal (MD) regions expressing AdipoR2 in wild-type mouse cortex. AdipoR2 is expressed by neurons in the DMHa (a, b) and MD (c, d) nuclei in aged WT mice at 48-52w. Images were acquired at 20x magnification. Scale bars for (a) and (c) are 200 µm and (b)–(d) are 50 µm.
